# Genome-Wide Search for Quantitative Trait Loci Controlling Important Plant and Flower Traits in Petunia Using an Interspecific Recombinant Inbred Population of *Petunia axillaris* and *Petunia exserta*

**DOI:** 10.1534/g3.118.200128

**Published:** 2018-05-15

**Authors:** Zhe Cao, Yufang Guo, Qian Yang, Yanhong He, Mohammed I. Fetouh, Ryan M. Warner, Zhanao Deng

**Affiliations:** * University of Florida, IFAS, Department of Environmental Horticulture, Gulf Coast Research and Education Center, Wimauma, FL 33598; † Department of Horticulture, Michigan State University, East Lansing, MI 48824; ‡ Key Laboratory of Horticultural Plant Biology, Ministry of Education, College of Horticulture and Forestry Sciences, Huazhong Agricultural University, Wuhan 430070, Hubei, China; § Faculty of Agriculture, Tanta University, Tanta, Postal Code 31527, Egypt

**Keywords:** Floral trait, *Petunia axillaris*, *Petunia exserta*, Quantitative trait locus, Recombinant inbred line

## Abstract

A major bottleneck in plant breeding has been the much limited genetic base and much reduced genetic diversity in domesticated, cultivated germplasm. Identification and utilization of favorable gene loci or alleles from wild or progenitor species can serve as an effective approach to increasing genetic diversity and breaking this bottleneck in plant breeding. This study was conducted to identify quantitative trait loci (QTL) in wild or progenitor petunia species that can be used to improve important horticultural traits in garden petunia. An F_7_ recombinant inbred population derived between *Petunia axillaris* and *P. exserta* was phenotyped for plant height, plant spread, plant size, flower counts, flower diameter, flower length, and days to anthesis in Florida in two consecutive years. Transgressive segregation was observed for all seven traits in both years. The broad-sense heritability estimates for the traits ranged from 0.20 (days to anthesis) to 0.62 (flower length). A genome-wide genetic linkage map consisting of 368 single nucleotide polymorphism bins and extending over 277 cM was searched to identify QTL for these traits. Nineteen QTL were identified and localized to five linkage groups. Eleven of the loci were identified consistently in both years; several loci explained up to 34.0% and 24.1% of the phenotypic variance for flower length and flower diameter, respectively. Multiple loci controlling different traits are co-localized in four intervals in four linkage groups. These intervals contain desirable alleles that can be introgressed into commercial petunia germplasm to expand the genetic base and improve plant performance and flower characteristics in petunia.

A major bottleneck in plant breeding has been the much limited genetic base and much reduced genetic diversity in domesticated, cultivated agricultural crops (Tanksley and McCouch 1997; Zamir 2001). Identification and utilization of favorable gene loci or alleles from wild or progenitor species have been suggested as an effective approach to increasing genetic diversity and breaking this bottleneck. Enormous effort has been made in major agronomic and horticultural crops to collect, preserve and characterize wild germplasm, identify favorable genes and alleles through genetic mapping, and introgess them into elite germplasm (Dempewolf *et al.* 2017). Nevertheless, little effort has been made or reported in ornamental plants.

The modern cultivated garden petunia (*Petunia ×hybrida*) is one of the most economically important ornamental plants. The wholesale value of garden petunia, including plants grown in flats, pots and hanging baskets, in the U.S.A. in 2014, was estimated at over $120 million (U.S. Department of Agriculture-National Agricultural (USDA) Statistics Service 2015). The main strategy used in developing petunia cultivars has relied on individual selection in segregating populations from the crosses among elite cultivars or breeding lines (Ewart 1981). However, strong selection driven by petunia breeders and hybridization of closely related breeding lines had resulted in high levels of similarity and low levels of genetic diversity among commercial petunias cultivars, which was associated with a major decline in consumer preference for petunia in the 1990s (Anonymous 1995; Griesbach 2006).

Utilization of wild species can be an effective approach to expand the germplasm and increase the diversity in garden petunia. Experiences in other crops have shown that many alleles representing broad genetic diversity and phenotypic variation reside in underdeveloped wild germplasm (Tanksley and Nelson 1996). Previous studies in petunia suggested that wild petunia species could provide useful genetic resources for improving commercial petunia cultivars across a range of traits (Griesbach *et al.* 2002; Walworth and Warner 2009; Warner and Walworth 2010). Their results have showed that *P. axillaris* and *P. integrifolia* possess superior alleles controlling fast development of successive leaves or nodes, *P. axillaris* carries freezing tolerance alleles, and *P. exserta* has early flower production alleles (Walworth and Warner 2009; Warner and Walworth 2010).

On the other hand, wild species often perform poorly in one or more horticultural aspects compared to commercial cultivars. As modern cultivars continue to diverge from their wild relatives, the use of wild germplasm for favorable allele introgression increasingly carries the penalty of introgressing many unwanted traits simultaneously (linkage drag) (de Vicente and Tanksley 1993). Breaking linkage drag requires intensive selection, which can be extremely difficult and time-consuming. Underscoring this point, Tanksley and Nelson (1996) indicated that a single gene from a wild species could drag with it sizeable chromosomal regions containing more than 100 inferior genes that even 20 years of traditional breeding cannot recombine out. Genetic linkage maps and QTL information have been used to monitor and facilitate gene transfer from wild germplasm to elite cultivars in many important agronomic crops (Zamir 2001). Tanksley and Nelson (1996) indicated that such information can substantially reduce linkage drag by at least tenfold compared with traditional breeding. In tomato, for example, genetic linkage maps have been employed to screen progeny carrying minimal donor chromosomal segments linked with disease resistance genes introgressed from wild germplasm (Tanksley and Nelson 1996).

So far, several genetic linkage maps have been developed in petunia based on restriction fragment length polymorphism (RFLP) (Strommer *et al.* 2000), amplified fragment length polymorphism (AFLP) (Galliot *et al.* 2006), and simple sequence repeat (SSR) markers (Klahre *et al.* 2011; Vallejo *et al.* 2015). The marker density in these linkage maps differed from an average of 10.2 cM between markers to an average of 3.18 cM between markers (Galliot *et al.* 2006; Vallejo *et al.* 2015). Based on these maps, several QTL have been identified in wild petunia species (Galliot *et al.* 2006; Klahre *et al.* 2011; Hermann *et al.* 2015). Most of these QTL studies centered around pollination syndrome-related traits such as pistil and stigma length, flower scent, and flower size. Only one QTL study focused on plant development rates and flowering times (Vallejo *et al.* 2015).

Recently, advances in next-generation sequencing (NGS) technologies have brought an efficient, low-cost, and large-scale marker discovery method that was referred as Genotyping-by-Sequencing (GBS) (Davey *et al.* 2011). A vast number of SNP markers generated by GBS have been used for linkage map construction, QTL analysis, and association analysis in many agronomic crops (He *et al.* 2014). However, in ornamental plants, including petunia, the use of GBS in linkage map construction and QTL analysis have lagged far behind agronomic crops (Vallejo *et al.* 2015).

The objectives of this study were to phenotype seven important quantitative traits, including plant height, plant spread, plant size, flower diameter, flower length, flower counts and days to anthesis, in a petunia F_7_ segregating population derived from an interspecific hybridization between *P. axillaris* and *P. exserta*, and to identify and localize QTL controlling these plant and flower traits based on a SNP marker-based linkage map.

## Materials and Methods

### Plant materials and field experiments

An F_7_ population comprising 173 recombinant inbred lines (RILs) was produced by single seed descent from an interspecific cross between *P*. *axillaris* (PI 28546; USDA Ornamental Plant Germplasm Center, Columbus, OH) and *P*. *exserta* [kindly provided by Dr. Robert Griesbach, USDA-Agriculture Research Service (ARS), Beltsville, MD] at Michigan State University (Guo *et al.* 2017). *Petunia axillaris* is a progenitor species of the modern commercial petunia (*P. × hybrida*), whereas *P. exserta* is a recently discovered species with interesting plant and flower characteristics (Griesbach *et al.* 1999).

Two field experiments were conducted in 2014 and 2015, from Jan. to July following the same growing calendar in both years, at the University of Florida’s Gulf Coast Research and Education Center (UF/GCREC) in central Florida. In early Jan., seeds of *P. axillaris* and *P. exserta* as well as RIL progeny were sown into 20-row germination trays (27.94 cm × 30.48 cm) filled with Fafard germination mix (Conrad Fafard, Inc., Agawam, MA, USA) and germinated in a growth room with room temperature maintained at 27° and light intensity at 150 μmol m^-2^ s^-1^. Two weeks later, germination trays with young seedlings were transferred to a greenhouse where the seedlings were grown on a metal bench, with air temperature maintained between 25° and 30° and a photoperiod of 16 h natural light and 8 h dark. Twelve days later, eight or more seedlings per RIL line were transplanted from the germination trays to 72-cell planter trays (66 cm × 33 cm) filled with Fafard 3B potting mix (Conrad Fafard, Inc.). Seedlings were fed twice a week with a water-soluble fertilizer containing 15% (w/w) total nitrogen, 5% phosphate (P_2_O_5_), and 15% potassium (K_2_O) (Peters Excel, Everris, USA). After two weeks, seedlings in planter trays were acclimated in a shade house with 30% light exclusion for one week. Then, four uniform seedlings per RIL and their parents were transplanted to mulched, raised ground beds equipped with an automated drip irrigation system at the GCREC experimental farm (N 27° 45”, W 82° 13”). The sandy-textured soil of the raised ground beds was fumigated with Pic-Clor 60 (60% chloropicrin and 40% 1, 3-dichloropropene) at 45 kg per 1,000 m^2^ one month prior to transplanting. After transplanting, each plant received 8 g of controlled release fertilizer (Osmocote, The Scotts Miracle-Gro Company, Marysville, OH, USA). The drip irrigation system ran 30 min daily. During the petunia growing season (late Feb. to mid-June), the daily average temperatures ranged from 11° to 28° in 2014, and from 6° to 28° in 2015. Total precipitation was 42.39 cm in 2014 and 46.30 cm in 2015. A randomized complete block design with four replicates was used for the field experiment each year.

### Phenotypic data collection and analysis

RIL population progeny and their parents were phenotyped for plant height (PH), plant spread (PS), plant size (PZ), flower diameter (FD), flower length (FL), flower counts (FC), and days to anthesis (DTA). The phenotypic data of PS and PH were collected near the end of the growing season (early to mid-June) to assess maximum plant growth potential. PS (cm) was calculated by averaging the maximal plant spread (cm) and minimal plant spread (cm). Maximal plant spread was measured along the longest axis of the plant from one edge to the opposite edge. Minimal plant spread was taken between plant edges perpendicular to maximal plant spread. PH was measured from the soil surface to the highest point of the plant. PZ was calculated using the formula: PZ = [π × (plant maximal spread ÷ 2) × (plant minimal spread ÷ 2) × plant height]. Three fully-opened fresh flowers were randomly selected from each plant for FD and FL measurements. FD was measured from one petal edge to the opposite edge of the petal, and FL was measured from the base of the calyx to the plane of the corolla. FC was recorded weekly (Krahl and Randle 1999) for seven weeks. DTA was calculated as the number of days from seed sowing to first anthesis.

The frequency distribution, Pearson’s correlation coefficients, and broad-sense heritability for each trait were analyzed using JMP Pro 10.0.2 (SAS institute, 2012). The statistical model for broad-sense heritability (*H^2^*) estimation was: *y_ijk_ = µ +* G*_i_ + E_j_ + G_i_ × E_j_ + B_h(j)_ + ε_ijk_*, where *y_ijk_* is the observed value of the studied trait, *µ* is the population mean, G*_i_* is genotypic effect, *E_j_* is environmental effect, *G_i_ × E_j_* is the interaction effect between genotype and environment, *B_h(j)_* is block effect, and *ε_ijk_* is random error. All effects were treated as random in the ANOVA. Broad-sense heritability was calculated as the proportion of the genotypic variance over the total phenotypic variance. The level of broad-sense heritability was categorized as low (*H*^2^ < 0.30), moderate (*H*^2^ between 0.30 and 0.60), or high (*H*^2^ > 0.60), according to the criteria proposed by Johnson *et al.* (1955).

### Linkage map construction and QTL analysis

A SNP-based genetic map (Guo *et al.* 2017) was used for QTL identification. The genetic linkage map consisted of 368 bins and covered a total of 277.1 cM across seven chromosomes. Putative QTL regions were detected by interval mapping (IM) and multiple QTL mapping (MQM) using MapQTL 6.0 (Van Ooijen 2004). Once QTL locations were determined by interval mapping, linked markers with the highest LOD scores were then treated as co-factors in the MQM model. The LOD score threshold for declaring QTL presence was determined by the 95^th^ percentile of LOD score from a permutation test (1000 cycles).

### Data availability

Phenotyping and genotyping data of all the petunia RILs used in this study, and their linkage mapping data are archived at Dryad (https://datadryad.org/resource/doi:10.5061/dryad.m23ks48). The original GBS data are available under the NCBI GenBank BioProject number PRJNA353949.

## Results

### Trait analysis

Mean values, ranges, and broad-sense heritability (*H*^2^) estimates for the seven traits studied are shown in Table 1. *Petunia axillaris* showed higher values than *P. exserta* in PS, PZ, FD, and FL, but had similar values with *P*. *exserta* in PH and FC for both years. *Petunia axillaris* and *P. exserta* displayed similar DTA values in 2014, but *P. exserta* had a higher DTA value than *P. axillaris* in 2015. DTA instability between 2014 and 2015 indicates high sensitivity of DTA to horticultural practices and/or environmental conditions.

**Table 1:** Phenotypic data of *P. axillaris*, *P. exserta*, and their recombinant inbred lines (RILs) for seven plant and flower traits when grown in central Florida, U.S.A. in 2014 and 2015, and the variance components and broad-sense heritability estimates for the seven traits

Trait	Year	Phenotypic data						Variance component estimates (%)					Broad-sense heritability*^b^*
		Parents			Mid parent value	RILs							
		*P. axillaris*	*P. exserta*	T-test*^a^*		Mean ± SD	Range (minimum to maximum)	*V_g_*	*V_e_*	*V_ge_*	*V_b_*	*ε*	
		Mean ± SD											
**PH (m)**	**2014**	0.51 ± 0.04	0.43 ± 0.06	ns	0.47	0.39 ± 0.12	0.12 - 0.76	60.67	0.48	6.61	0.05	32.19	0.61
	**2015**	0.52 ± 0.07	0.55 ± 0.11	ns	0.53	0.41 ± 0.12	0.10 - 0.77						
**PS (m)**	**2014**	1.10 ± 0.10	0.81 ± 0.09	*	0.95	0.74 ± 0.18	0.24 - 1.20	52.93	1.72	18.64	0	26.71	0.53
	**2015**	1.09 ± 0.08	0.76 ± 0.07	*	0.92	0.78 ± 0.26	0.11 - 1.41						
**PZ (m^3^)**	**2014**	0.15 ± 0.04	0.07 ± 0.03	*	0.11	0.19 ± 0.11	0.02 - 0.54	46.81	5.47	19.12	0	28.60	0.47
	**2015**	0.16 ± 0.05	0.09 ± 0.02	*	0.13	0.24 ± 0.17	0.01 - 0.81						
**FD (cm)**	**2014**	5.23 ± 0.05	4.12 ± 0.23	*	4.68	4.62 ± 0.59	2.55 - 6.30	50.22	6.05	11.10	2.07	30.57	0.50
	**2015**	5.12 ± 0.07	3.85 ± 0.13	*	4.49	4.42 ± 0.53	3.10 - 5.97						
**FL (cm)**	**2014**	4.98 ± 0.18	4.16 ± 0.19	*	4.57	4.68 ± 0.53	2.70 - 5.95	62.45	0.66	13.27	0.36	24.66	0.62
	**2015**	5.06 ± 0.14	4.23 ± 0.15	*	4.65	4.61 ± 0.48	3.00 - 5.93						
**FC (no.)**	**2014**	211.00 ± 52.05	194.33 ± 37.16	ns	202.50	165.41 ± 78.52	12.00 - 458.00	36.01	0	33.95	0.14	29.90	0.36
	**2015**	213.67 ± 26.35	276.00 ± 44.23	ns	244.50	164.78 ± 92.70	10.47 - 528.00						
**DTA (day)**	**2014**	75.33 ± 1.51	76.86 ± 2.68	ns	76.01	73.92 ± 6.94	57.00 - 105.00	20.49	0	59.49	0.14	19.88	0.20
	**2015**	76.00 ± 3.00	81.63 ± 2.20	*	78.81	74.66 ± 10.09	51.00 - 97.00						

PH, plant height; PS, plant spread; PZ, plant size; FD, flower diameter; FL, flower length; FC, flower counts; DTA, days to anthesis.

*V*_g_, *V*_e_, *V*_ge_, *V*_b_, and *ε* are variance components from genotype, environment, genotype by environment interaction, field block, and random error, respectively.

a Student’s t-tests were performed to compare means of traits between *P. axillaris* and *P. exserta* in 2014 and 2015. * significant at *P* < 0.05; ns not significant at *P* < 0.05.

b Broad-sense heritability, the proportion of *V_g_* in the total phenotypic variance.

**Figure 1:**
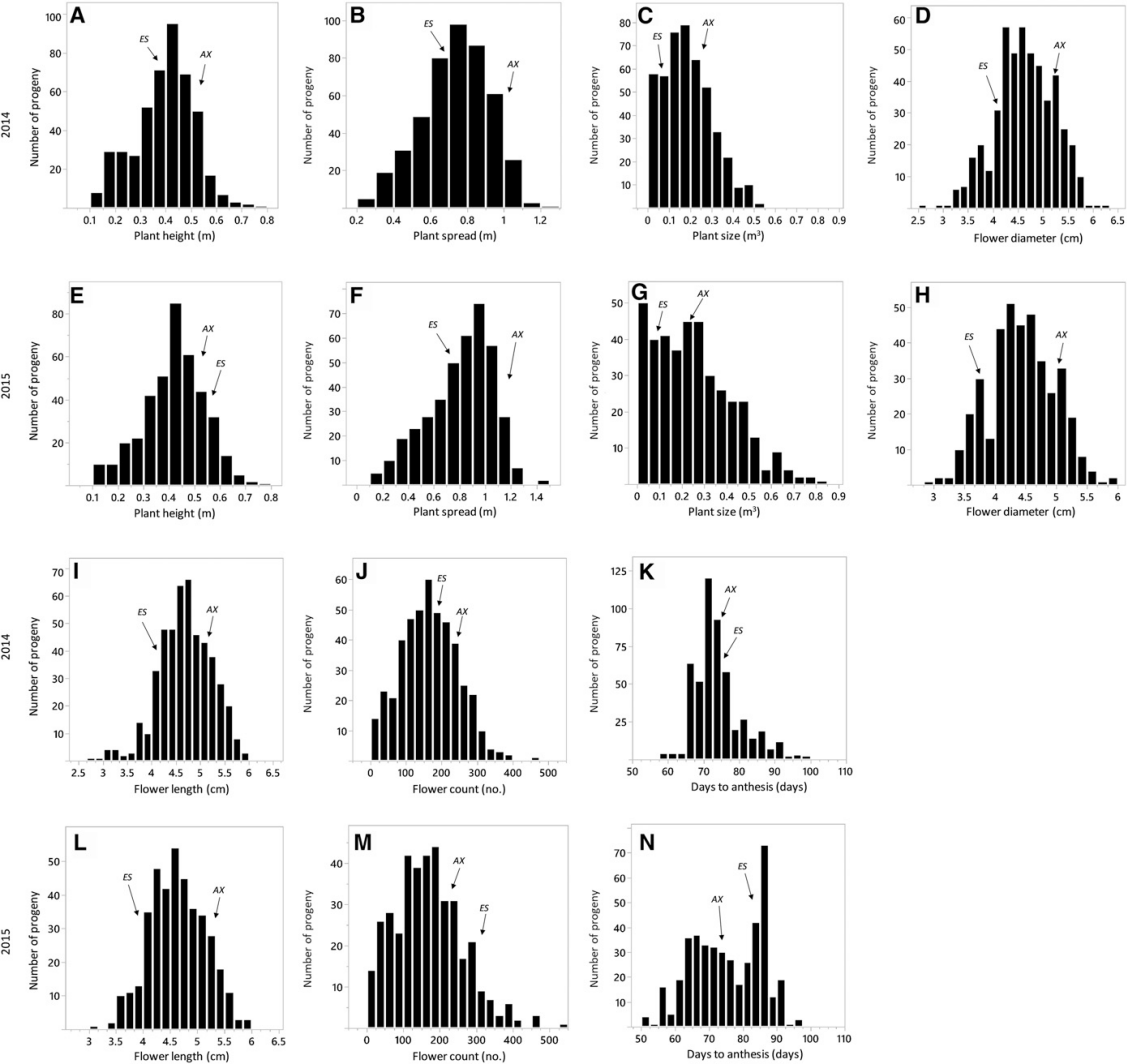
Frequency distribution of RIL progeny in a *P. axillaris* and *P. exserta* F_7_ population that was phenotyped for seven plant and flower traits (on the horizontal axis) in 2014 (first and third rows) and 2015 (second and fourth rows). ES and AX represent *Petunia exserta* and *P. axillaris*, respectively. A plant height (2014), B plant spread (2014), C plant size (2014), D flower diameter (2015), E plant height (2015), F plant spread (2015), G plant size (2015), H flower diameter (2015), I flower length (2014), J flower count (2014), K days to anthesis (2014), L flower length (2015), M flower count (2015), and N days to anthesis (2015).

The RIL population derived from *P. axillaris* and *P. exserta* exhibited transgressive segregation for all seven traits in both years (Table 1, Figure 1). Of the seven traits examined, high *H*^2^ values were observed for PH (*H*^2^ = 0.61) and FL (*H*^2^ = 0.62); moderate *H*^2^ estimates were for PS (*H*^2^ = 0.53), PZ (*H*^2^ = 0.47), FD (*H*^2^ = 0.50), and FC (*H*^2^ = 0.36), and low *H*^2^ value was for DTA (*H*^2^ = 0.20) (Table 1). As for variance components, the block effect (*V_b_*) for five traits ranged from 0.00% (plant size) to 2.07% (flower diameter), indicating that *V_b_* contributed negligibly to the total phenotypic variance (*V_p_*). The proportion of environmental components (*V_e_*) in the total phenotypic variance was lower for PS (1.72%), FC (0.00%), DTA (0.00%), and FL (0.66%), but higher for FD (6.05%) and PZ (5.47%) (Table 1). Considerable genotype (G) by environment (E) interaction variance (*V_ge_*) was seen for PZ (19.12%), PS (18.64%), FC (33.95%), and DTA (59.49%) (Table 1).

Pearson’s correlation analysis suggested that 10 of the 21 possible trait pairs had significant (*P <* 0.001) correlations (Table 2). Weak but significant correlations between floral and plant traits were found between FL and PH (*r*^2^ = 0.131), FL and PS (*r*^2^ = 0.149), FD and DTA (*r*^2^ = 0.114), FD and FC (*r*^2^ = 0.127), and FC and PH (*r*^2^ = 0.123). Three trait pairs, FD and FL (*r*^2^ = 0.435), FC and PS (*r*^2^ = 0.314), and PH and PS (*r*^2^ = -0.385), had a moderate correlation coefficient level, while two more trait pairs, PH and PZ (*r*^2^ = 0.653), and PS and PZ (*r*^2^ = 0.840), displayed relatively high correlation coefficients.

**Table 2:** Pearson’s correlation coefficients between traits phenotyped in an F_7_ population of *P. axillaris* and *P. exserta* in central Florida, U.S.A. in 2014 and 2015

Trait^z^	PH	PS	PZ	FD	FL	FC
**PS**	−0.385*					
**PZ**	0.653*	0.840*				
**FD**	0.093	0.071	−0.052			
**FL**	0.131*	0.149*	−0.056	0.435*		
**FC**	0.123*	0.314*	−0.087	0.127*	−0.124	
**DTA**	0.101	−0.033	0.069	0.114*	0.022	−0.021

PH, plant height; PS, plant spread; PZ, plant size; FD, flower diameter; FL, flower length; FC, flower counts; DTA, days to anthesis.

* significant at *P* < 0.001.

### QTL analysis

**Figure 2:**
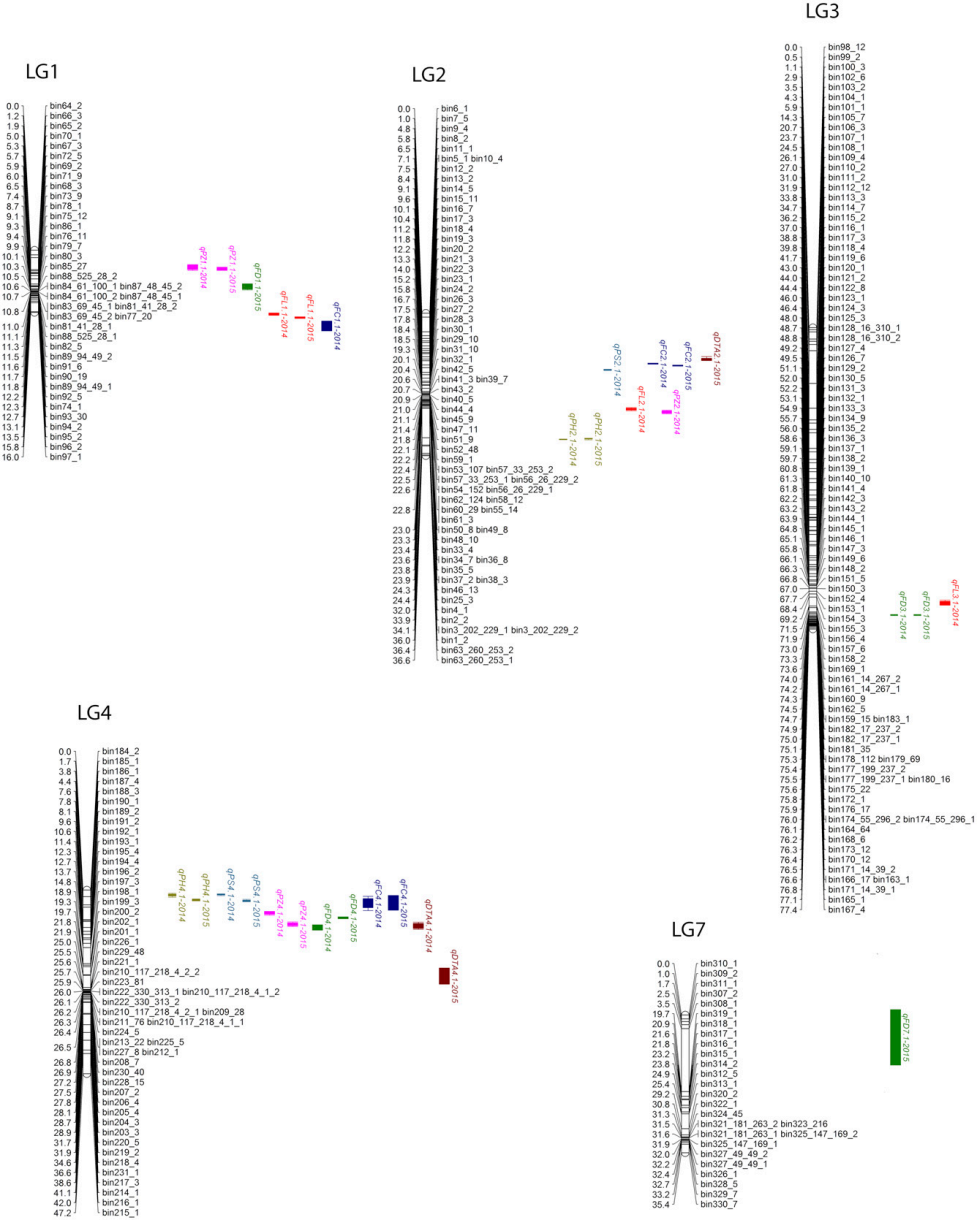
Genetic linkage map and position of QTL for seven plant and flower traits identified in a *P. axillaris* and *P. exserta* F_7_ population. SNP marker bins are listed on the right side of the linkage groups (LG), and the corresponding genetic distances based on recombination rates are shown on the left side of the linkage groups. Locations of QTL are shown by names and colored bars to the right of the linkage groups.

In this study, the LOD threshold values determined by permutation tests were either 2.7 for FD, FL, FC in both years, and for PZ in 2015, or 2.8 for PH and PS for both years, and for PZ in 2014. A summary of QTL controlling seven petunia traits identified in 2014 and 2015 is shown in Table 3 and Figure 2.

**Table 3:** Summary of QTL identified in an F_7_ population of *P. axillaris* and *P. exserta* for seven plant and flower traits, and the position, additive effect, and percentage of phenotypic variance explained by the QTL

Trait	Year	QTL	LOD	LG	Nearest marker	Position	Additive effect	% PVE	LOD threshold
PH	2014	*qPH2.1*	9.00	2	bin4_1	31.990	−7.73	22.9	2.8
		*qPH4.1*	3.43	4	bin185_1	1.674	3.04	9.6	
	2015	*qPH2.1*	5.58	2	bin4_1	31.990	−6.49	14.5	2.8
		*qPH4.1*	3.86	4	bin185_1	1.696	3.69	10.7	
PS	2014	*qPS2.1*	3.38	2	bin24_3	15.846	−0.05	8.7	2.8
		*qPS4.1*	6.33	4	bin185_1	1.674	0.07	17.1	2.8
	2015	*qPS4.1*	6.68	4	bin185_1	1.696	10.12	19.6	
PZ	2014	*qPZ1.1*	3.01	1	bin66_3	1.220	0.03	6.8	2.8
		*qPZ2.1*	3.69	2	bin16_7	10.141	−0.03	8.4	
		*qPZ4.1*	7.34	4	bin185-1	1.696	0.04	17.8	
	2015	*qPZ1.1*	2.86	1	bin75_12	9.126	0.04	7.8	2.7
		*qPZ4.1*	4.62	4	bin188_3	7.352	0.06	13.1	
FD	2014	*qFD3.1*	5.75	3	bin178_112	75.254	0.20	13.3	2.7
		*qFD4.1*	9.77	4	bin191_2	9.600	0.28	24.1	
	2015	*qFD1.1*	4.46	1	bin68_3	7.344	0.11	9.1	2.7
		*qFD3.1*	4.19	3	bin179_69	75.300	0.15	8.6	
		*qFD4.1*	5.46	4	bin188_3	7.560	0.17	11.5	
		*qFD7.1*	5.21	7	bin317_2	12.479	0.21	10.6	
FL	2014	*qFL1.1*	10.22	1	bin85_27	10.283	0.24	22.5	2.7
		*qFL2.1*	8.48	2	bin25_3	28.381	−0.29	16.2	
		*qFL3.1*	4.48	3	bin159_15	74.661	0.15	10.6	
	2015	*qFL1.1*	11.22	1	bin92_5	12.153	0.26	34.0	2.7
FC	2014	*qFC1.1*	5.64	1	bin95_2	14.583	−25.66	12.3	2.7
		*qFC2.1*	7.14	2	bin52_48	22.113	−29.13	16.1	
		*qFC4.1*	8.66	4	bin185_1	1.696	32.31	20.1	
	2015	*qFC2.1*	3.75	2	bin51_9	21.693	−30.51	12.0	2.7
		*qFC4.1*	3.60	4	bin185_1	1.696	29.85	12.1	
DTA	2014	*qDTA4.1*	4.07	4	bin192_1	10.553	3.00	12.6	2.7
	2015	*qDTA4.1*	5.21	4	bin197_3	16.785	3.78	12.9	2.7
		*qDTA2.1*	3.80	2	bin59_1	21.798	−3.07	9.2	

PH, plant height; PS, plant spread; PZ, plant size; FD, flower diameter; FL, flower length; FC, flower counts; DTA, days to anthesis.

PVE, percentage of variance explained.

#### Plant height (PH):

Two putative QTL controlling PH were identified in linkage group (LG) 2 (*qPH2.1*) and LG4 (*qPH4.1*) (Table 3). Both QTL were consistently detected in both years. The locus *qPH2.1* was a major QTL explaining 22.9% and 14.5% of the phenotypic variation in 2014 and 2015, respectively. The QTL *qPH4.1* explained much less phenotypic variance, 9.6% in 2014 and 10.7% in 2015.

#### Plant spread (PS):

Two putative QTL in LG2 (*qPS2.1*) and LG4 (*qPS4.1*) were identified for PS (Table 3). The QTL *qPS4.1* was consistently detected in 2014 and 2015. The percentage of phenotypic variance explained (PVE) by *qPS4.1* was 17.1% in 2014 and 19.6% in 2015. The locus *qPS2.1* was detected only in 2014, and its PVE was 8.7%.

#### Plant size (PZ):

The putative QTL *qPZ1.1*, *qPZ2.1*, and *qPZ4.1* controlling PZ were detected in LG1, LG2, and LG4, respectively; the loci *qPZ1.1* and *qPZ4.1* were consistently evident in both years with the former explaining 6.8% in 2014 and 7.8% in 2015, and the latter explaining 17.8% in 2014 and 13.1% in 2015 (Table 3). The locus *qPZ2.1* was only detected in 2015, and its PVE was 8.4%.

#### Flower diameter (FD):

Four putative QTL controlling FD were identified in LG1 (*qFD1.1*), LG3 (*qFD3.1*), LG4 (*qFD4.1*), and LG7 (*qFD7.1*) (Table 3). Among them, *qFD3.1* and *qFD4.1* were detected in both years, while *qFD1.1* and *qFD7.1* were detected in only 2015. The PVE of these QTL varied from 8.6% (*qFD3.1*, in 2015) to 24.1% (*qFD4.1*, in 2014). The PVE of *qFD*4.1 varied in two years, 24.1% in 2014 and 11.5% in 2015.

#### Flower length (FL):

Three putative QTL in LG1 (*qFL1.1*), LG2 (*qFL2.1*), and LG3 (*qFL3.1*) were identified for this trait (Table 3). The locus *qFL1.1* was detected consistently in both years, while *qFL2.1* and *qFL3.1* were detected only in 2014 and 2015, respectively. Locus *qFL1.1* showed the largest effect, explaining 22.5% of the phenotypic variance in 2014 and 34.0% in 2015.

#### Total flower count (FC):

Three putative QTL located in LG1 (*qFC1.1*), LG2 (*qFC2.1*), and LG4 (*qFC4.1*) were detected controlling FC (Table 3). The QTL *qFC2.1* and *qFC4.1* were significant in both 2014 and 2015, while *qFC1.1* was observed only in 2014. The PVE of these QTL ranged from 12.0% (*qFC2.1*, 2015) to 20.1% (*qFC4.1*, 2014).

#### Days to anthesis (DTA):

Two putative QTL in LG2 (*qDTA2.1*) and LG4 (*qDTA4.1*) were detected controlling DTA (Table 3). The QTL *qDTA4.1* was localized to the same position in both years, and its PVEs in both years were similar, 12.6% in 2014 and 12.9% in 2015. The QTL *qDTA2.1* was observed only in 2015 and had a lower PVE of 9.2%.

## Discussion

Recently, interests in introgressing useful alleles from *P. exserta* to cultivated *P. ×hybrida* have been strong (Griesbach *et al.* 1999; Watanabe *et al.* 2001; Walworth and Warner 2009). Phenotypic data from this study showed that *P. exserta* was similar to *P. axillaris* in PH and FC, but was smaller than *P. axillaris* in PS, PZ, FD, and FL. These results suggest *P. exserta* could develop more flowers than *P. axillaris* per given unit area. *Petunia exserta* appears to carry desirable alleles for enhanced canopy coverage while *P. axillaris* likely possesses superior alleles for larger-sized flowers, both highly prized traits in cultivar development.

For all seven traits examined in both years, there was evident transgressive segregation in the F_7_ population of *P. axillaris* and *P. exserta*. Extreme phenotype values on both sides of the distribution were observed for all traits studied, even for FC where both parents showed similar phenotypic values. Observation of wide transgressive segregation was also reported for DTA, FD, and FL in the F_2_ population of *P. axillaris* × *P. exserta* (Warner and Walworth 2010). These results indicate multiple genes are involved for all seven traits studied.

Broad-sense heritability (*H*^2^) estimates in petunia for PH (*H*^2^ = 0.83 to 0.88), DTA (*H*^2^ = 0.34 to 0.88), FD (*H*^2^ = 0.72 to 0.82), and FL (*H*^2^ = 0.72 to 0.96) were previously reported by others (Hussein and Misiha 1979; Warner and Walworth 2010; Vallejo *et al.* 2015). Compared to those *H*^2^ estimates, the present study reports relatively lower *H*^2^ for PH (*H*^2^ = 0.61), FD (*H*^2^ = 0.50), FL (*H*^2^ = 0.62), and DTA (*H*^2^ = 0.20) (Table 1). The lower *H^2^* estimates might be due to the partitioning of genotype × year interactions from genotypic component on a two-year-based heritability calculation (Burton and Devane 1953). Should genotypes be evaluated in a single location for one year, the *H^2^* estimates tend to be biased upward as the G × E component would be integrated into the genetic component (Dudley and Moll 1969). The *H*^2^ values of petunia FC (*H*^2^ = 0.36), PS (*H*^2^ = 0.53), and PZ (*H*^2^ = 0.47) were first reported in this study. In other ornamental species, similar or higher *H*^2^ values for FC were reported in *Dimorphotheca pluvialis* (*H*^2^ = 0.29) (Hof *et al.* 1999), daylily (*H*^2^ = 0.67) (Fogaça *et al.* 2012), and rose (*H*^2^ = 0.74) (Liang *et al.* 2017), and a lower *H*^2^ value for PZ was observed in *Viola sororia* (*H*^2^ = 0.36) (Antlfinger *et al.* 1985). The lower *H*^2^ for petunia FC observed in this study indicated that selection for higher FC in progeny of *P. axillaris* × *P. exserta* may be less effective than in other plants with higher *H*^2^ values for flower count.

In this study, the correlation between FC and PS (*r*^2^ = 0.314) was higher than FC and PH (*r*^2^ = 0.123), indicating that petunia RILs with larger PS values tended to develop more flowers than RILs with higher PH values. And the negative correlation between PS and PH (*r*^2^ = -0.385) suggested that the stem elongation in petunia plants tended to grow either vertically or horizontally. The correlation coefficient between FD and FL (*r*^2^ = 0.435) was moderate, suggesting that flowers with larger diameters tend to be longer. Floriculturally speaking, larger and longer flowers would have greater aesthetical impact and contribute positively to the commercial and landscape value of new petunia cultivars. Weak but significant correlations were observed between FD and FC (*r*^2^ = 0.127), and between FD and DTA (*r*^2^ = 0.114), which were similar to previous results observed in an F_2_ population of *P. axillaris* and *P. exserta* (Warner and Walworth 2010). The positive correlation between FD and FC suggests large-flowered petunia genotypes do not necessarily need to kill flower counts; instead they may have the potential to produce more flowers, which is of significant value for future petunia breeding.

A high-density linkage map is an important prerequisite for identification and localization of QTL controlling quantitative traits. In this study, we used a SNP-based genetic map consisting of 368 bins and covering 277.1 cM across the seven petunia chromosomes. Compared to previously reported petunia genetic linkage maps, the resolution of this map has been substantially refined to 0.75 cM between bins (Galliot *et al.* 2006; Klahre *et al.* 2011; Vallejo *et al.* 2015), thus providing a very useful tool for fine mapping of important QTL. However, bins were unevenly distributed across seven linkage groups (Figure 2). Taking LG4 for example, 25 bins were concentrated within a 5.3-cM interval (23.15 – 28.48 cM), while the other 18 bins spanned a larger interval of 41.50 cM. Similar uneven distribution of molecular markers was reported by Klahre *et al.* (2011) for the linkage map of petunia chromosomes 2, 5, and 7 based on a *P. axillaris × P. inflata* F_2_ population. Strommer *et al.* (2002) reported low recombination frequencies in a *P. × hybrida* RIL population, resulting in the clustering of AFLP markers and also short maps of petunia linkage groups. Robbins *et al.* (1995) also reported extensive restricted recombination occurring in wild petunia hybrids as revealed by T-DNA insertions. The observed/reported recombination restrictions of interspecific petunia hybrids will likely result in linkage drag when attempting to introgress useful alleles from wild petunia species into commercial petunia cultivars.

The effectiveness of QTL analysis largely depends on how accurately the target traits can be phenotyped in the mapping population (Cobb *et al.* 2013). Under natural field conditions, heritability estimates for many quantitative traits usually drop to less than 50% (Kearsey 1998). RIL populations can be a powerful tool for detecting low-heritable QTL as each RIL genotype can be replicated across different environments and phenotyped multiple times to reduce environmental effects (Broman 2005). In the present study, a total of 19 QTL were identified by phenotyping the RIL population in multiple field blocks over two years. Eleven of the QTL (57.9%) were consistently identified in both years, including two QTL for PH, one QTL for PS, two QTL for PZ, two QTL for FD, one QTL for FL, two QTL for FC, and one QTL for DTA. Several consistently detected QTL could be regarded as major QTL that explain major proportions of the total phenotypic variance, including *qPH2.1* (PVE = 22.9% in 2014 and PVE = 14.5% in 2015), *qFL1.1* (PVE = 22.5% in 2014, and 34.0% in 2015), and *qFD4.1* (PVE = 24.1% in 2014, and 11.5% in 2015). These major QTL could be valuable for further QTL and gene discovery and thus for petunia breeding.

Co-localization of QTL was observed in several intervals of four petunia linkage groups, including five QTL controlling PS, PZ, FD, FL, and FC in a segment of LG1 (7.3 cM to 14.6 cM), and three QTL controlling PH, PS, FC, FL, and DTA in an interval (21.7 cM to 31.9 cM) within LG2 (Figure 2). Previously, Vallejo *et al.* (2015) observed a cluster of five QTL controlling flower diameter, flower length, node development rate, number of lateral branches, and number of flower buds in a segment of LG1 (17.0 cM to 24.0 cM). Co-localized QTL were also reported in rice, sorghum and *Brassica napus* (Brondani *et al.* 2002; Ding *et al.* 2012; Zou *et al.* 2012). The presence of QTL-rich chromosomal segments might be due to close genetic linkage of genes, gene pleiotropism, or restricted recombination. The availability of petunia whole genome sequences (Bombarely *et al.* 2016) may help to identify the corresponding physical intervals and genes located in these QTL-rich chromosomal regions.
